# Polymorphism of hydrogen-bonded star mesogens – a combinatorial DFT-D and FT-IR spectroscopy study†

**DOI:** 10.1039/c8ra09458f

**Published:** 2019-03-14

**Authors:** Michael Pfletscher, Janek Wysoglad, Jochen S. Gutmann, Michael Giese

**Affiliations:** Institute of Organic Chemistry, University Duisburg-Essen Universitätsstr. 7 45141 Essen Germany michael.giese@uni-due.de; Institute of Physical Chemistry, University Duisburg-Essen Universitätsstr. 2 45141 Essen Germany; Institute of Physical Chemistry, CENIDE, University Duisburg-Essen Universitätsstr. 2 45141 Essen Germany

## Abstract

A comprehensive study combining detailed computational analyses with temperature-variable FT-IR experiments was performed in order to elucidate the structure of the hydrogen-bonded liquid crystals based on phloroglucinol and azopyridine in their mesophase. Conformational analysis revealed three relevant conformers: star, λ- and E-shape. The results demonstrate an entropy-driven unfolding mechanism of the assembly. The stability of the conformers is given by intermolecular π–π and dispersion interactions of the azopyridine side chains. Correlating the calculated vibrational frequency with experimental FT-IR spectra suggests a λ-folded conformation of the assemblies as the predominant species in the mesophase.

## Abstract 2

The structure of hydrogen-bonded star mesogens is investigated using modern quantum chemistry methods in combination with infrared spectroscopy.

## Graphical abstract

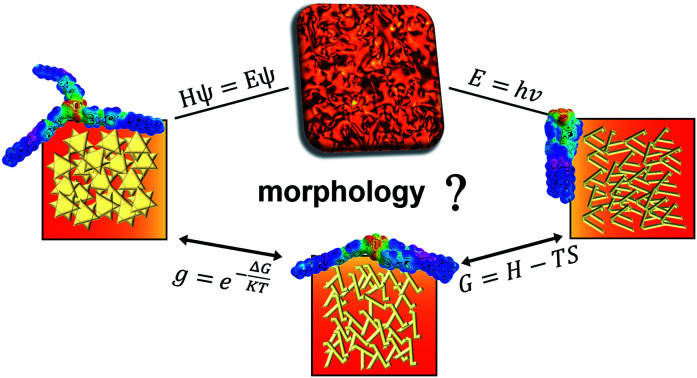

## Introduction

The design of new materials such as polymers,^1^ gels,^2^ liquid crystals,^3^ requires a detailed understanding of the structure–property relationships, which are ruled by a complex interplay of intermolecular forces. An attractive approach to these so-called “smart materials” is molecular self-assembly by employing specific non-covalent interactions such as hydrogen or halogen bonding.

**Fig. 1:**
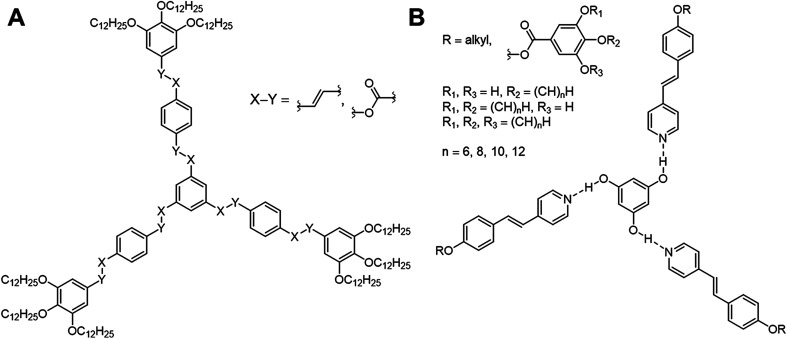
Representative three-armed mesogens, which can appear in a covalent (A) and supramolecular (B) system. These representatives occur in different shapes and form thus specific mesophases depending on the linker (A, X–Y) and peripheral alkyl groups (B).

However, the design of non-conventional^4,5^ and hydrogen-bonded^6–10^ liquid crystals (LCs) has become an attractive research area within the past decades. For instance, three-armed mesogens also called “Hekates”^11^ (Fig. 1A) are able to form columnar mesophases *via* nanosegregation of the rigid core units and flexible alkyl chains, which makes these soft materials interesting for electronic applications, such as in organic semiconductors or for applications in photovoltaic cells.^12^ Depending on connectivity (X–Y), they form shape-persistent or semi-flexible LC stars with varying molecular shapes (star (S), lambda (λ) and E-folded shape), which differ tremendously in their self-assembly behaviours and material properties.^11,13–15^

Although these systems have been well investigated,^13,14,16–21^ only few examples of hydrogen-bonded star mesogens are known.^22–27^ Within the past two decades seminal works by Kato, Fréchet, Lehn and Percec^7–9,28–37^ have demonstrated that self-assembled LCs have a broad application potential in scientific and technological fields due to mimicking the efficient self-assembly process of nature.^38–45^ In contrast to their covalent pendants, supramolecular star mesogens are more flexible and may show more complex mesostructures (see for instance Fig. 1B). Since subtle changes in their architecture have a tremendous impact on the geometries and physical properties of the hydrogen-bonded liquid crystals, a precise prediction of their structure–property relationships is challenging.^19,46^ Lee *et al.* demonstrated that hydrogen-bonded star mesogens occur in two different morphology depending on the number and substitution pattern of the peripheral alkoxyl groups. They found smectic or columnar arrangements of the star mesogens.^25–27^

**Fig. 2:**
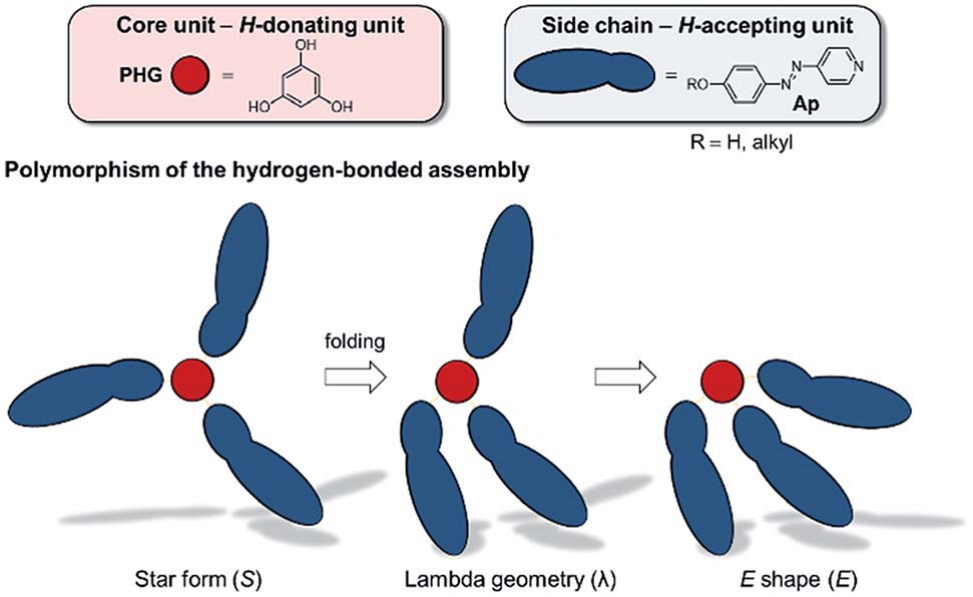
Graphical illustration of the polymorphism of three-armed mesogens in a hydrogen-bonded system based on PHG and Ap used as H-donating and H-accepting unit, respectively.

Since 2016, our group has started detailed studies on the structure–property relationships of hydrogen-bonded mesogens (Fig. 2). Based on a modular approach combining hydrogen bond donating core units (phloroglucinol, PHG) with hydrogen bond accepting side chains (azopyridine, Ap), we reported a variety of liquid crystalline materials with photo-switchable properties, which exhibited fast and reversible phase transitions (nematic N → isotropic I) upon irradiation with a commercially available laser pointer (405 nm, 5 mW).^22,46^ Applying this concept, we investigated the impact of the core^46^ and side chain on the liquid crystalline behaviour of these assemblies. For instance, the degree and pattern of fluorination control the nature of the mesophase (nematic or smectic) and enhance the temperature range of liquid crystalline phase.^47^ While the fluorination on the PHG core unit tends to stabilise the nematic phase, mono- and difluorination on the arenes of the azopyridine component cause a change of the order of the mesogens, proving the impact of fluorination on the properties of supramolecular LCs. In another study, the linker group between the pyridyl and phenylene moieties was varied, which led to a variety of different stable mesomorphic phases,^19^ ranging from nematics to different smectic mesophases. While the PHG-based aggregates prefer smectic alignment when the side chains are linked by olefin bridges, the change of the linking group to thio-/oxo-ester and azo group yields a nematic phase.

Based on solid state structures we already suggested a linear arrangement of our hydrogen-bonded mesogens by a partly folding mechanism into a λ-folded geometry (Fig. 2).^22,24,46^ However, so far we did not investigate the shape of the hydrogen-bonded assemblies in their mesophase.

The present study aims to gain insight into the structural conformation of the three-armed hydrogen-bonded mesogen by combining dispersion-corrected density functional theory (DFT-D) calculations with temperature-variable infrared spectroscopy (t-FT-IR).

## Results and discussion

### Concept and conformational analysis

Based on the data obtained from crystallographic and small-angle X-ray scattering of the hydrogen bonded assembly with phloroglucinol, the results indicate a lambda geometry instead of the star-shaped mesogen as proposed by Lee *et al.*^25–27^ The aim of the present study is to clarify the question of the molecular morphology of the investigated three-armed hydrogen-bonded assembly within their mesophase by employing a combination of DFT-D calculations and t-FT-IR spectroscopy. Since Foresman and Frisch^48,49^ reported a balanced compromise between accuracy and computational cost for the APF-D^49^ density functional approximation Řezáč^50^ suggested the use of the 6-31G basis sets with additionally polarisation functions for calculations of non-covalent systems. Herein, we are using the APF-D hybrid functional including dispersion correction term with the widely used standard basis set 6-31G(d) of Pople^51^ as our standard model chemistry.

Hydrogen bonds play a vital role in supramolecular systems, since it provides a high flexibility of molecular shape. Most hydrogen-bonded mesogens previously reported rely on the interaction between benzoic acid derivatives and pyridines, forming rod-shaped dimers either by homo association between the acid molecules^52–54^ or by heterogeneous association of benzoic acid with pyridine.^45,55^ For the latter, the shape is stabilized by additional interaction between the 2*H* of the pyridine with the carbonyl oxygen atom.^56–64^ In contrast to these binding motifs, the interaction between phenol (PH) and pyridine (pyr) is significantly weaker leading to a higher flexibility and enhances structural diversity.^46,65–67^

In order to find a suitable starting geometry for our study, we initially investigated the PH⋯pyr interaction by conformational analysis (details see ESI, Chapter 3†). Hereby, we obtained 121 optimized conformer geometries, which differ in their energies (zero point energy ZPE and Gibbs free energy G; ESI Fig. S1 and S2†). Systematic analyses of ZPE of 121 different conformers of our PH⋯pyr model demonstrate the flexibility of a phenolic-pyridine system leading to a high structural diversity.

**Fig. 3:**
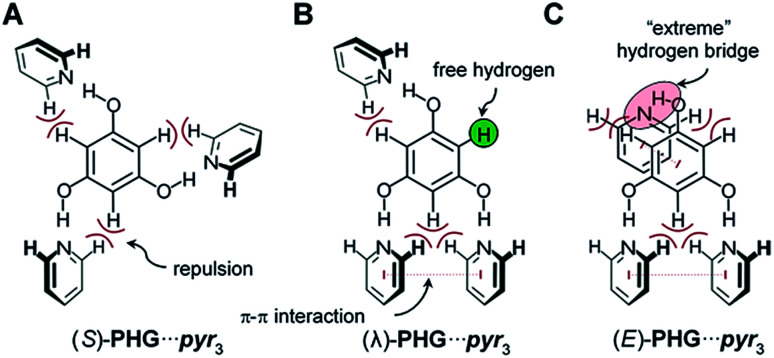
Three main conformations of PHG⋯pyr_3_ auxiliary investigated within this study. Depending on the order of the pyridines, different intramolecular forces occur between phloroglucinol core and pyridine side chains.

Based on the conformational analysis of PH⋯pyr three starting conformations, the S-, λ- and E-folded forms have been identified for the PHG⋯pyr_3_ auxiliary (Fig. 3).

### Structural analysis of phloroglucinol-based auxiliary (PHG⋯pyr_3_)

These three conformers are well-known for covalent Hekates,^68^ but have not been investigated for hydrogen-bonded systems based on PHG and pyr. Most star conformers with *C*_3_-symmetry show large void space between the three pyridine arms, which needs to be filled by alkyl chains of surrounding molecules or by folding of the side chains (yielding λ and E-shape, respectively) to guarantee a dense packing in the condensed state.^68^ The folding of the mesogenic structure is supported by intramolecular π–π and CH–π interactions between the core and side chain units.

For the identification of the favoured hydrogen-bonded conformer in this truncated model, DFT studies were carried out using APF-D/6-31G(d) level of theory. In order to prove that the method chosen is suitable for the structural elucidation of PHG⋯pyr_3_ auxiliary, we performed additional calculations using semi-local density functional B97-D3 ^69–71^ and the evidently most popular hybrid functional B3LYP^72,73^ with Grimme's dispersion correction including Becke–Johnson damping.^69,70^ B3LYP and B97-D3 have been used for many studies of non-covalent interactions^74–79^ and liquid crystalline materials.^80,81^ In addition to our standard basis set 6-31G(d), we also applied the extended polarized and augmented (added diffuse functions) triple zeta basis set 6-311+G(2d,p) to evaluate the impact of basis sets on optimized geometries, energies and vibrational frequencies. Most calculations were additionally performed with counterpoise correction (CP).^82,83^

Comparing ZPE of the three basic conformers calculated at 25 °C indicates a preference for the (λ)-PHG⋯pyr_3_ conformation (lowest ΔZPE), whereas the star conformer (S)-PHG⋯pyr_3_ revealed highest ΔZPE (Table 1 and ESI Table S1†). The higher ΔZPEs of the E-folded PHG⋯pyr conformer using CP method indicate a violent overlapping of “ghost orbitals” caused by the stacking pyr units. Accordingly, that leads to a higher basis set superposition error (BSSE).^82,84^ The obtained angles and lengths of hydrogen bridges of optimized geometries (ESI Table S2–S4†) are in good agreement to experimental results.^22,24,46^ In order to get a better understanding of the dynamics of the conformer distribution the Gibbs free energies of the PHG⋯pyr_3_ conformers have been calculated for different temperatures by applying the Boltzmann weighting method (Fig. 5 and ESI Table S1†).^85^

**Table 1:** Conformational population distribution (pop.) and relative Gibbs free energies (Δ*G*) of PHG⋯pyr_3_ auxiliary at different temperatures using APF-D method with basis sets 6-31G(d), 6-31G(d) CP and 6-311+G(2d,p)

*T*	Conformer	Basis sets					
		6-31G(d)		6-31G(d) CP		6-311+G(2d,p)	
		Δ*G* (kJ mol^−1^)	Pop. (%)	Δ*G* (kJ mol^−1^)	Pop. (%)	Δ*G* (kJ mol^−1^)	Pop. (%)
25 °C	(λ)	0.0	66.4	2.3	28.6	0.7	43.2
	(E)	15.2	0.1	21.8	0.0	13.2	0.3
	(S)	1.7	33.5	0.0	71.4	0.0	56.6
75 °C	(λ)	0.0	49.9	3.6	22.4	1.2	39.6
	(E)	17.2	0.1	24.5	0.0	14.5	0.4
	(S)	0.0	50.0	0.0	77.6	0.0	60.0
90 °C	(λ)	0.5	45.7	4.0	21.0	1.7	36.2
	(E)	18.2	0.1	25.3	0.0	15.2	0.4
	(S)	0.0	54.2	0.0	78.9	0.0	63.3
120 °C	(λ)	1.5	38.4	4.8	18.8	2.6	30.7
	(E)	20.4	0.1	26.9	0.0	16.6	0.4
	(S)	0.0	61.4	0.0	81.2	0.0	68.9

**Fig. 4:**
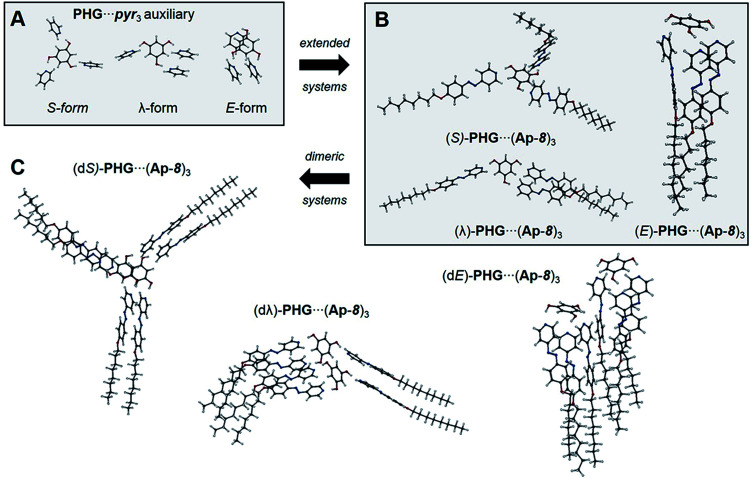
Optimized structural geometries of PHG⋯pyr_3_ auxiliary (A in the S-, λ- and E-shape), monomers (B) and dimers (C) of PHG⋯(Ap-8)_3_ assemblies.

**Fig. 5:**
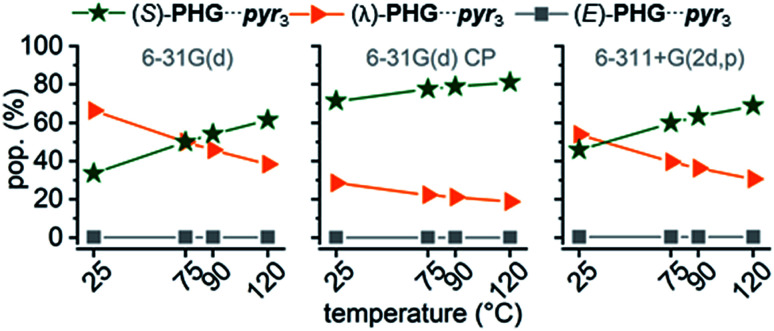
Temperature dependence of conformational population distribution (pop.) of PHG⋯pyr_3_ auxiliary using APF-D method with basis sets 6-31G(d), 6-31G(d) incl. CP correction and 6-311+G(2d,p).

The results indicate that an increase of temperature yields a destabilization of (λ)-PHG⋯pyr_3_ and a more increased population of (S)-PHG⋯pyr_3_, while the (E)-conformer seems to play a minor role in the investigated temperature range (less than 1%, Fig. 5). These findings can be attributed to lower entropy contribution to Gibbs free energy at higher temperatures (see Table 1). The population of the different conformation is consistent within the APF-D method, which is attributed to the chosen APF-D functional considering repulsion and attraction both at intermediate and long distances.^49^ In contrast, using B3LYP-D3 and B97-D3 level of theory reveals some inconsistencies in the in the population of the most likely conformers depending on the employed basis set (ESI Fig. S3†). Superimposition of PHG⋯pyr_3_-based conformers regarding different basis sets with and without CP reveals a critical displacement of atoms for basis set 6-31G(d) without CP. Computations using CP and costly basis set 6-311+G(2d,p) show however virtually identical structural geometries (ESI Fig. S4†). The three conformations of PHG⋯pyr_3_ yielded completely different vibrational profiles (ESI Fig. S5†). The most significant differences were found for the aromatic ring deformation (Rβ) of the pyridyl group in the range of 1750–1480 cm^−1^. This characteristic vibrational pattern can be used for the identification of the PHG⋯(Ap-8)_3_ polymorphs. Since a variety of vibrations overlap in the range of lower wavenumbers, our discussion will focus on the vibrations in the range of 1750–1480 cm^−1^. A detailed discussion of the vibrational frequencies of the PHG⋯pyr_3_ auxiliary can be found in the ESI (Section 4†).

Due to BSSE accompanied by a tremendous increase of the number of atoms within the PHG⋯(Ap-8)_3_ system, the three conformers of this assembly were obtained including CP (Fig. 4B). In addition, we performed calculations of PHG⋯(Ap-8)_3_ dimers (Fig. 4C) to consider IR spectral changes by interactions with neighbouring assemblies. These dimers are denoted with “d” (*e.g.* (dS)-PHG⋯(Ap-8)_3_). Considering the computational cost, these calculations have been performed without CP.

### DFT-D calculations of PHG⋯(Ap-8)_3_ monomers and dimers

The computation of a hydrogen-bonded system with larger side chain units ensures the consideration of further intra-/intermolecular interactions (*e.g.* π–π-stacking, dispersion), which control the supramolecular architecture. Therefore, we applied the APF-D/6-31G(d) level of theory with CP correction, to calculate the monomeric PHG⋯(Ap-8)_3_ assembly in the S-, λ- and E-folded shapes. In general, the calculations show a crucial influence on the BSSE: for the E-folded conformation the error of the ground state energy was reflected by −70.3 kJ mol^−1^, which corresponds to 20.4% of the uncorrected ZPE. Hence, the ZPEs of λ- and S-shaped conformation was overestimated by −47.3 kJ mol^−1^ (18.1%) and −25.4 kJ mol^−1^ (15.1%) respectively.

In contrast to the auxiliary, the calculations of the three conformations of PHG⋯(Ap-8)_3_ at 25 °C indicate a preference for the E-folded conformation (Table 2). This can be attributed to the strong stabilization (complexation energy *E*_C_ ∼ −302 kJ mol^−1^) of the long side chains *via* CH–π forces and dispersion, which will be discussed in the following. In the E-folded analogue the hydroxyl groups are strongly twisted with respect to the PHG plane (up to 42°, ESI Table S8†), which affects the length of the hydrogen bonds. Two hydrogen bonds were found to be approximately ∼2.0 Å and the remaining corresponds to ∼1.9 Å (Table 2). Although the E-folded PHG assembly revealed the lowest ZPE, we assume that the assembly is not preferred due to the strong tension of the hydrogen bonds, leading to a decay of the assembly. In contrast, the λ- and S-shaped conformers of PHG⋯(Ap-8)_3_ do not show any strained or stretched hydrogen bridges.

**Table 2:** Lengths (*D*) and angles (*ζ*) of hydrogen bridges, complexation energies (*E*_C_), ΔZPE, Δ*G* and average population distribution (pop.) of the PHG⋯(Ap-8)_3_ conformers using APF-D/6-31G(d) level of theory with CP correction

	*D*(H⋯N) (Å)	*ζ*(O–H⋯N) (°)	*E*_C_ (kJ mol^−1^)	ΔZPE (kJ mol^−1^)	Δ*G*^b^ (kJ mol^−1^)	pop^b^ (%)
(E)	2.045	137.2	−302.2	0.0	0.0	98.7
	1.912^a^	153.7			0.3	42.8
	1.883^a^	149.9			5.3	9.0
(λ)	1.857	167.3	−213.6	57.0	10.7	1.3
	1.886^a^	168.6			0.0	46.8
	1.869^a^	170.7			0.0 (0)	45.7
(S)	1.852	167.7	−143.5	113.3	25.1	0.0
	1.854	166.6			4.6	10.4
	1.850	168.6			0.0 (2)	45.4

a Parameters obtained from the two parallel side chains in the head-to-head fashion.

b Gibbs free energies and conformer populations are listed from top to bottom: 25, 90 and 120 °C.

In particular, the λ-folded conformer is mainly stabilized by π–π-interaction and dispersion^22,46^ of the two parallel oriented side chains (*E*_C_ ∼ −214 kJ mol^−1^). This optimized geometry corresponds nearly to the molecular structure observed in the solid phase.^46^ Regarding the non-folded star conformer, no intramolecular forces between the side chains were observed explaining the high value of ΔZPE. The hydrogen bridges in the S- and λ-conformers do not differ significantly referring to lengths and angles. A brief overview of relevant computed geometries and energies of the conformers is given in Table 2. More detailed information is available in the ESI (ESI Table S8†). Vibrational frequency calculations of the three optimized PHG⋯(Ap-8)_3_ conformers at elevated temperatures yielded a strongly preferred E-shape conformation (Table 2). Since we are interested in identifying the molecular shape of the PHG aggregates within the nematic and isotropic phase, we calculated in addition the Gibbs free energy at 90 °C as well as 120 °C. Based on the Boltzmann weighting distribution with respect to different temperatures clearly shows that population of (λ)-PHG⋯(Azp-8)_3_ is preferred at elevated temperatures (∼47%), while the population of the E-folded PHG conformer is decreasing to ∼9% (Table 2). Interestingly, the unfolded star conformer appears in the same quantity with the λ-folded conformer in the isotropic state. These findings indicate that the polymorphism of these hydrogen-bonded stars can be preserved by introducing energy (increased temperature) into the supramolecular system leading to an isomerisation of PHG⋯(Azp-8)_3_ from E- → λ-folded → S-unfolded conformations (see Fig. 2).

Since the liquid crystallinity is also driven by the electronical anisotropy of mesogens, we calculated the electronical properties of these conformers using the same level of theory. Moreover, the conformations differ in their polarizability and dipole moment (ESI Table S6†). While the dipole moment decreases in the series S → λ → E, the polarizability increases in the same direction. This is in line with the calculated electrostatic surface potential (ESI, Fig. S3†). Likewise, an identical distribution of conformers as well as trends for polarizability and dipole moment was obtained using B97-D3/def2svp^86^ level of theory (ESI Table S6†).

In order to identify the dominant conformation of the hydrogen-bonded system by their vibrational modes, we studied also dimeric system for each conformer. The design of dimers was chosen by employing the following criteria: within the star dimers the S-assembly was stacked so that high segregation occurs among the aromatics and alkyl chains, which corresponds to a columnar mesostructure. The dimer based on the λ-folded structure was inspired by the solid state structure of PHG⋯(Ap-6)_3_.^46^ According to the *d*-spacing obtained by X-ray scattering of PHG⋯(Ap-8)_3_ the two E-folded structures were arranged side by side to reach a longitudinal distance of ∼35 Å representing the director length within the nematic phase. The result of the dimer computation is visualized in Fig. 4C. The investigation of the dimers led to similar results as reported for the isolated PHG⋯(Ap-8)_3_ assemblies and no significant structural differences were found. Vibrational frequency calculations however revealed differences between the isolated assembly and the dimers, which will be discussed in the final section of the manuscript.

### Interaction energy analysis of the conformational star assemblies

Dispersion forces between non-covalently bonded molecules or building blocks are crucial to stabilize the mesogenic structure as well as segregation processes to form highly ordered mesophases.^87–89^ Although many DFT and molecular dynamics studies exist for small covalent LC systems,^80,90–97^ giving reasonable explanation of their structural diversity, a computational study of hydrogen-bonded system has not been reported so far. To get a deeper understanding of such supramolecular materials those studies would be highly valuable.

Since the population analysis of the three conformers of PHG⋯(Ap-8)_3_ suggests an unfolding mechanism driven by the temperature, single point energy (SPE) calculations using counterpoise method have been performed. While *E*_C_ is directly obtained by CP method, we estimated further interaction energies (Δ*E*_int_). For the determination of Δ*E*_int_, single fragments were systematically removed by ∼10 Å and/or twisted (details see ESI Section 5.3†). We are well aware that synergetic effects prevail making the assignment of Δ*E*_int_ difficult and to some extend inaccurate. Nevertheless, we can obtain approximate values that explain the stability of these conformers.

Based on the PHG conformer study, the complexation energies were lowered within the series star, λ- and E-folded structures (−143.5 → −213.6 → −302.2 kJ mol^−1^). The number of interactions was enhanced after each folding. The first flip of an azopyridine side chain gave an energy gain of 70.1 kJ mol^−1^, whereas the flip of the second azopyridine, to obtain the E-shaped conformation, lowered the ZPE by −88.6 kJ mol^−1^. The higher energy drop calculated to the last scaffold is attributed to the interaction of all three side chains *via* CH–π-, π–π- and van der Waals interactions. To evaluate the individual interaction contributions in the two folded conformers, we performed SPE calculations, where the peripheral *O*-alkyl groups in the azopyridine side chains were partly (for λ-folded) or fully (for E-folded) *O*-methylated (see ESI, Table S10 and 11,† green highlighted structures). The reduction of the alkyl chain length allows to consider the energetic contribution of the π–π-interactions of the side chains. However, the complexation energies were lowered to −188.0 and −234.2 kJ mol^−1^ for the *O*-methylated λ- and E-folded structures, respectively (ESI Table S10 and 11†). Correlating the complexation energies of the lambda geometries with *O*-octyl and *O*-methyl chain yielded 25.6 kJ mol^−1^ for the van der Waals interaction (ESI Table S10†). Since the folding from the star to the λ-folded structure of PHG⋯(Ap-8)_3_ yielded 70.1 kJ mol^−1^, we can thus estimate an Δ*E*_int_ of 44.5 kJ mol^−1^ for the π–π stacking. This corresponds virtually to the Δ*E*_int_ obtained from further SPE calculations (ESI Table S11†).

Due to the complex architecture of the E-shaped assembly, we calculated interaction energies based on different SPE calculations of E-folded geometries, where fragments have been removed selectively from each other (see ESI Table S12†). According to this, we determined an interaction energy of 41.6 kJ mol^−1^ for the π–π stacking. The two azopyridines, which are aligned to the third azo group in an edge-to-face fashion, cause CH–π interactions of 64.3 kJ mol^−1^. The dispersion interaction between the aliphatic chains was basically estimated by the difference of Δ*E*_C_ of the *O*-octyl- and *O*-methyl-based aggregates. This corresponds to 67.8 kJ mol^−1^ (thus 22.6 kJ per mol per octyl chain) and is in-line with results obtained for the λ-folded assembly.

**Fig. 6:**
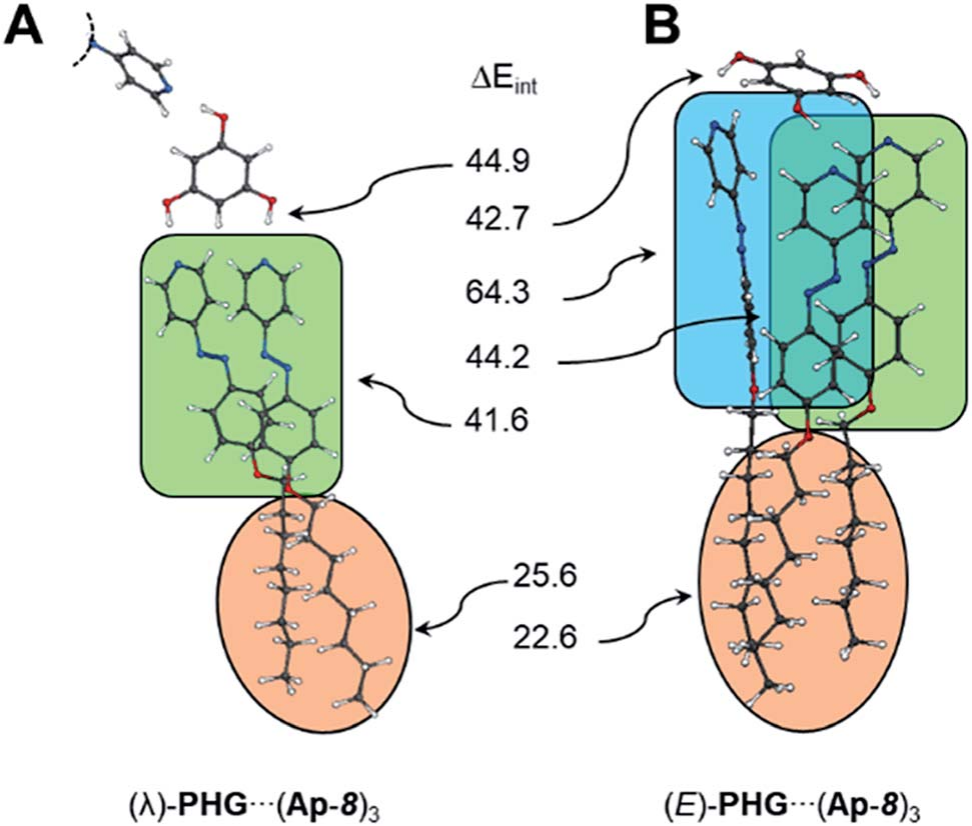
Overview of the interaction energies (kJ mol^−1^) calculated in the λ- and E-folded conformations. Orange: dispersion, green: π–π interaction, blue: CH–π forces.

An overview of the calculated interaction energies in the folded conformers is given in Fig. 6. The strength of the hydrogen-bridge, for the unfolded star assembly, was estimated to ∼47.0 kJ mol^−1^, which is insignificantly higher than for the λ-folded conformer (∼44.9 kJ mol^−1^). The E-shaped assembly yields interaction energies for the hydrogen bridges of ∼42.7 kJ mol^−1^. These are in-line with the results obtained in the conformational analysis study. A detailed discussion of the interaction energies can be found in the ESI (ESI Table S9–S11†).

The analysis of the individual contributions of the interactions clearly shows their impact on the stabilization of the folded structures. Although the azopyridines in (E)-PHG⋯(Ap-8)_3_ contribute most to the folded stability, we assume that the assembly is not preferred due to the strong tension of the hydrogen bonds, leading to a decay of the assembly, particularly at elevated temperatures.

### Vibrational frequency correlation with experimental IR data

**Fig. 7:**
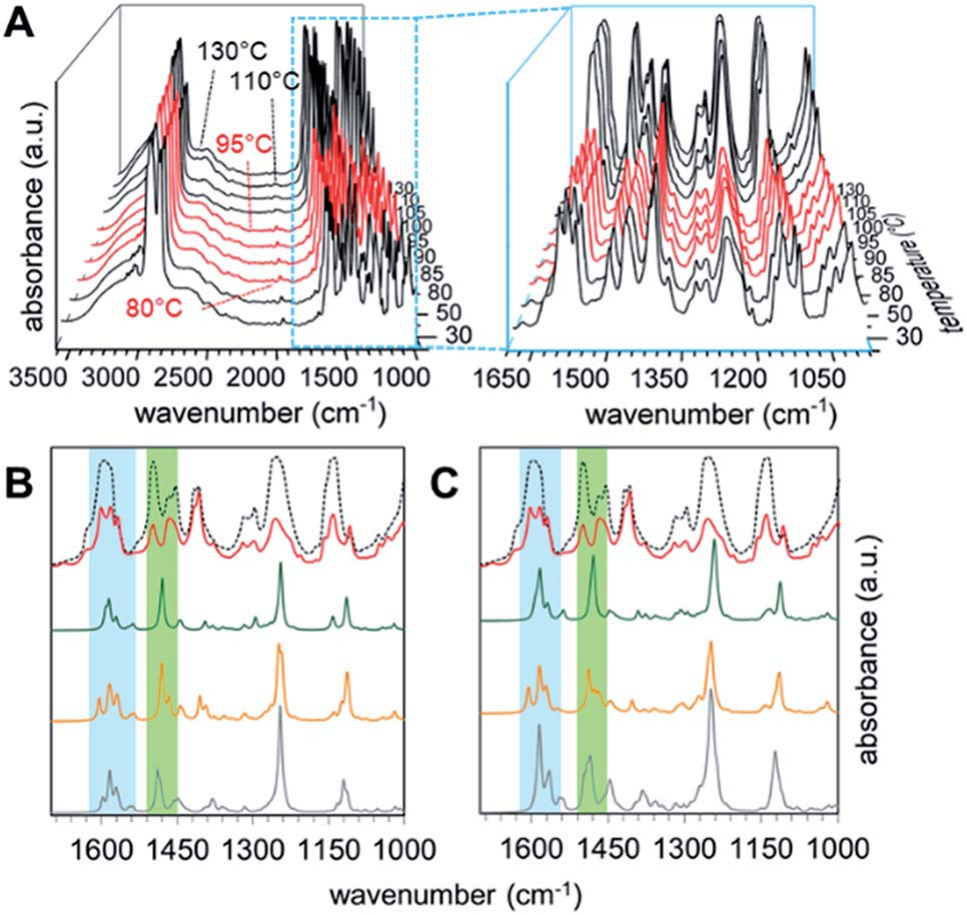
Experimental temperature-variable IR spectra of PHG⋯(Ap-8)_3_ (A) and calculated IR spectra of related monomers (B) and dimers (C) assemblies. For (B) and (C) spectra of S- (green), λ- (orange) and E-shaped (grey) conformations are plotted coheres with 130 °C (black doted) and 90 °C (red) experiment. Blue and green areas highlight interesting regions in the IR spectra showing quadrant ring and semicircle stretches.

In order to get an insight into the structure of the PHG⋯(Ap-8)_3_ assembly in the mesophase, vibration frequency calculations of the three conformers were performed and correlated with experimental findings by t-FT-IR spectroscopy (Fig. 7). The structural differences should be especially observed near to the core unit due to additional repulsive proton interaction or void spaces (Fig. 3), which will be accompanied with changes in the vibrational frequency modes of these conformers.

The simulated IR spectra for the PHG⋯(Ap-8)_3_ conformers and their dimers show three peaks in the range of 1620–1550 cm^−1^ (see ESI, Chapter 5.4†), except for the E-shaped dimer. A closer look into these vibrations reveals ring deformation signals of the benzenes in the pyridyl units, which are responsible for a characteristic peak splitting for the different PHG⋯(Ap-8)_3_ conformers (ESI Table S12†). While the in-plane bending (β) of the hydroxyl groups occurs at ∼1595 cm^−1^ in the S- and E-shaped conformers/dimers, it is shifted to higher frequency in the λ-folded conformer/dimer. A pair of vibrations at higher frequency was merely obtained for the E-folded dimer. The intensity of one of these vibrations in the region 1620–1550 cm^−1^ is more increased, due to ring deformation of the outer benzene cores. The intense vibrations at 1280–1210 cm^−1^ observed in all conformers and dimers are explained by the combinatorial vibrations from ring deformation and rocking of the benzene as well as by the aliphatic groups, respectively.

Correlation of the experimental IR data of the PHG⋯(Ap-8)_3_ assembly measured between 25 and 130 °C (Fig. 7A) with the simulated IR spectra for the three conformers allows to identify the predominant conformer within the mesophase. Upon cooling from the isotropic melt reveals changes of the vibrational spectra starting at 95 °C, which correlates to the isotropic–nematic transition obtained on DSC (Fig. 7A).^22^ The IR spectra within the crystalline (below 80 °C, Fig. 7A, black curves) and the liquid crystalline phases (Fig. 7A, red curves) appear almost identical, indicating similar structural morphologies of the assembly. The hydrogen bond between PHG and the pyridyl moieties remains intact even at elevated temperatures (until 130 °C, black curves), as proven by the broad band between 3200–2500 cm^−1^ (Fig. 7A). An increase of the intensities for nearly all vibrations in the region of lower frequency denotes changes in the environment and are associated with phase transitions, where groups gather in more polar areas.^98–101^ The experimental IR spectra show a set of three bands (1598, 1581 and 1567 cm^−1^), which are indicative for the ring deformation vibrations of three different pyridyl units.^102^ Furthermore, this is accompanied by three different semicircle stretches of the pyridyl units in the range 1500–1410 cm^−1^.^102^ The nematic nature of the assembly is reflected by the vibrations observed in the range 1286–1201 cm^−1^, representing twisting-rocking of methylene groups.^102^

Comparing the calculated spectra of the monomeric and dimeric assemblies of all three conformers with the experimental IR data of PHG⋯(Ap-8)_3_ obtained at 90 °C reveals that the λ-folded conformer seems to be the dominant species in the nematic phase. This observation is in-line with the results obtained in the population analysis of the monomeric PHG⋯(Ap-8)_3_ conformers. In the range of 1620–1550 cm^−1^, the characteristic vibration pattern calculated for the (S)-PHG⋯(Ap-8)_3_ assembly as well as their dimers does not match the experimental infrared spectra (Fig. 7B and C, blue area). Therefore, the presence of this conformer in the nematic phase can be ruled out. The shape and position of these vibration bands (1598, 1581 and 1567 cm^−1^) in the experimental IR spectra, however, give the hint that the PHG⋯(Ap-8)_3_ assembly adopts a λ-shaped geometry within the nematic phase (Fig. 7B and C, blue area). Although, the three vibrations can also be found in the calculated IR profile in the monomeric (E)-PHG⋯(Ap-8)_3_ system, these vibrations are vanished in the E-dimer calculated (Fig. 7B and C), whereas the E-folded conformer can be rule out.

The semicircle stretches between 1510–1455 cm^−1^ further supports the existence of a λ-folded morphology (Fig. 7C, green area). The findings are consistent with results obtained from the single crystal structures based on azopyridines and stilbazoles.^22,46^

In addition, the correlation of simulated and measured IR data suggests that the conformeric structure remains intact within the nematic phase, although the three hydrogen-bonds in the PHG⋯(Ap-8)_3_ introducing a degree of flexibility. This is in contrast to the Hekate analogs, which can fold due to highly flexible spacer groups (oxo-ester bridge) and form complex discotic phases.^11^ The stability of these discotic phases is given by neighbouring molecules, forming a screw-like superstructure, whereas the flexibility of such hydrogen-bonded three-armed mesogens seems to be limited by the void space required here. Depending on the OH⋯N binding motif, a partial folding of two side chains *via* intermolecular π–π forces and dispersions take place, forming the lambda structure. An unfolding of the individual building blocks occurs only after phase transition to the isotropic state, which is demonstrated by the changes of the semicircle ring deformation mode at ∼1500 cm^−1^ (Fig. 7B and C, black doted curves). Comparing the vibrations from the experimental with the calculated spectra within the blue and green labeled regions gives a good approximation of the vibrations obtained from the S-unfolded conformer. We assume that the λ-folded analogue still exists somewhat in the isotropic state, since some characteristic vibrations can be observed within the region of the semicircle stretches (Fig. 7B and C, green area).

However, further comparisons of the IR spectra are difficult, since vibrations out of these ranges are strongly overlaid with other vibration modes.

## Conclusions

In summary, the structure of hydrogen-bonded star mesogens based on phloroglucinol and azopyridines was investigated by a combination of density functional theory calculations and temperature-variable infrared spectroscopy.

Based on two models, PH⋯pyr and PHG⋯pyr_3_, we identified three fundamental conformations: S-, λ- and E-folded PHG conformers. Computations using different level of theory show that the APF-D/6-31G(d) method including counterpoise correction provides reliable results for these hydrogen-bonded systems and can be applied for other supramolecular systems. Conformational population distribution analysis at relevant temperatures and a correlation of the vibrational frequencies of optimized monomeric and dimeric PHG⋯(Ap-8)_3_ conformer geometries with experimental IR spectra revealed a preference of the λ-folded PHG conformer even at elevated temperature. This is in-line with existing crystallographic data^22,24,46^ and stays in contrast to previous reports by Lee *et al.* who reported on a closely related hydrogen-bonded system.^25–27^ Additionally, the results indicate that hydrogen-bonded star mesogens seem to persist crystalline-nematic phase transitions shown by IR spectra. Further studies were accomplished *via* counterpoise method and single point energy calculations, to explore the stability of the various conformers.

The present study provides a deep insight into the complex mesomorphic behavior of hydrogen-bonded materials and will facilitate the design of novel assemblies with specific liquid crystalline properties.

## Conflicts of interest

The authors declare no conflict of interest.

## Supplementary Material

RA-009-C8RA09458F-s001
