# Supplementary material for: Polymorphism of hydrogen-bonded star mesogens – a combinatorial DFT-D and FT-IR spectroscopy study
Source: RSC Adv. 2019 Mar 14;9(15):8444–53. doi: 10.1039/c8ra09458f (PMC9061708; doi:10.1039/c8ra09458f)
Supplement: RA-009-C8RA09458F-s001 [file RA-009-C8RA09458F-s001.pdf]

Electronic Supplementary Material (ESI) for RSC Advances.
This journal is © The Royal Society of Chemistry 2019

Supporting Information for

Polymorphism of Hydrogen-bonded Star Mesogens – A Combinatorial DFT-D and FT-IR Spectroscopy Study

Michael Pfletscher,[a†] Janek Wysoglad,[b†] Jochen S. Gutmann[c] and Michael Giese[*a]

[a]Institute of Organic Chemistry, University Duisburg-Essen, Universitätsstr. 7, *D*-45141 Essen. E-mail: michael.giese@uni-due.de

[b]Institute of Physical Chemistry and CENIDE, University Duisburg-Essen, Universitätsstr. 2, *D*-45141 Essen

[c]Institute of Physical Chemistry and CENIDE, University Duisburg-Essen, Universitätsstr. 2, *D*-45141 Essen

[†]Michael Pfletscher and Janek Wysoglad contributed equally to this work

[*]Corresponding author

Index

1	Computational Chemistry	3
2	Temperature-variable FT-IR Spectroscopy	3
3	Conformational Analysis (CA)	4
4	Computational Chemistry of the **PHG**···***pyr***$_3$ Auxiliary	6
4.1	Population Distribution Analysis	6
4.2	Geometric Parameters	9
4.3	Vibrational Modes	11
5	Characteristic Values within the **PHG**···(**Ap-8**)$_3$ System	12
5.1	Electronic Properties and calculated Energies	12
5.2	Geometric Parameters	14
5.3	Complexation and Interaction Energies	15
5.4	Vibrational Modes	16
6	References	18

1 Computational Chemistry

Within this study, all DFT calculations were performed using the Gaussian16[1] software package. Default values for the self-consistent-field (scf=tight) and geometry optimization convergence criteria as well as a default integration grid for numerical integrations (int=ultrafine) have been used. Full geometry optimization of **PHG···*pyr***$_3$ conformers were computed with density functional approximations (DFAs) B97-D3[2–4], B3LYP[5,6], APF-D[7] and two standard types of basis sets 6-31G(d)[8] and 6-311+G(2d,p), respectively. All DFAs comprise London dispersion correction term: B97-D3 and B3LYP-D3 were specified with Grimme's D3 dispersion correction and Becke-Johnson (BJ) damping.[2,3] APF-D uses a different empirical dispersion correction that is based on a spherical atom model.[7] Geometry optimizations and single point energy (SPE) calculations of **PHG···(Ap-*8*)**$_3$ model systems were calculated using the APF-D/6-31G(d) level of theory. Due to the basis set superposition error (BSSE), an erroneous effect of overlapping basis functions that is usually most significant with small basis sets, the computations of the **PHG···(Ap-*8*)**$_3$ monomer assembly were carried out with counterpoise correction[9,10] (CP). Subsequent to geometry optimizations, vibrational frequency calculations were accomplished at the same level of theory to characterize the true energy minima on the potential energy surface. For energetic comparison of conformers, the calculated zero-point energy (ZPE) and Gibbs free energy (G) are the deciding thermodynamic quantity for optimized structures, respectively. Regarding the conformation population analysis, Gibbs free energies were calculated for 298, 363, 348 and 393 K. For plots of calculated infrared spectra a full width at half maximum (FWHM) of 4 cm^{-1} was used. GaussView6[11] was utilized to verify normal mode assignments and to create electron density cube files. For visualisation of the optimized structures Mercury[12] 3.8 were utilized. Electrostatic potential isosurfaces (ESP) were given by MoleCoolQt64[13]. Based on the suggestion of Bader *et al.*[14], we employed a ρ(r) = 0.001 au (electrons bohr^{-3}) contour, encompassing roughly 97% of the molecules' electronic charge. Superimposition of Molecules have been performed using Maestro[15] software package (Schrödinger Suites).

2 Temperature-variable FT-IR Spectroscopy

The individual units **Ap-8** and **PHG** were mixed in the molar ration of 3:1 in acetone and the solvent was evaporated under reduced pressure. A sample was then prepared as a thin film between two CaF_2 single crystals, and the prepared sample was placed between two thermally conductive metal plates and heated up to 130 °C. After equilibration of the sample within the isotropic phase for some seconds, the temperature was reduced stepwise (increment of 5 °C) and an FT-IR spectrum (Bruker Vertex 70, He-Ne Laser, λ = 632 nm) was recorded.

3 Conformational Analysis (CA)

Relaxed potential energy surface (PES) computations were carried out on a phenol/pyridine system (**PH**···***pyr***) by scanning angle ζ(C−O···N) and dihedral angle Φ(C=C−O···N) stepwise. Using density functional theory APF-D level of theory with the basis set 6-31G(d) ensured a correlation of the energetic and geometric parameters with all calculated geometries obtained within the study. It should be noticed that the PES computation is visualized using uncorrected energy values and therefore molecular translational, rotational and vibrational constituents for enthalpy and entropy are not considered. The CA was performed by changing angles ζ and Φ by ~10° for ten steps each with the result that a computation grid of 11x11 points was obtained.

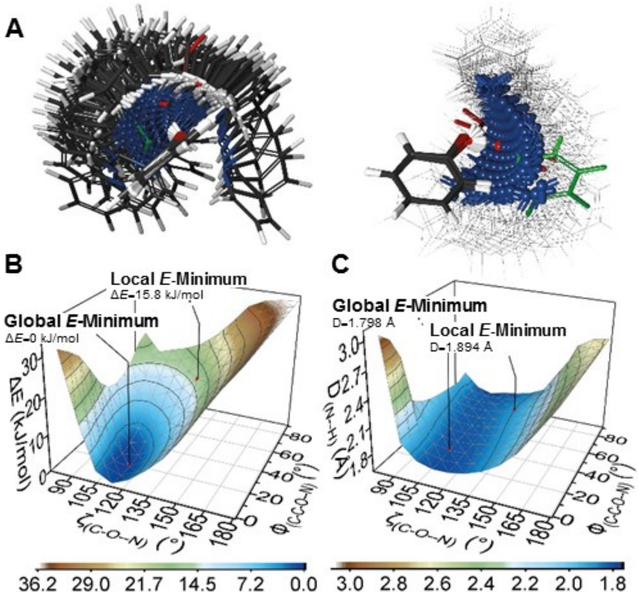

Supporting Figure S1. All 121 optimized geometries (A) and the corresponding relaxed PESs (B and C) of the **PH**···***pyr*** system are illustrated, showing the energetic change of the hydrogen bridge based on the relative position of pyridine to O–H group. The hemispherical-like interaction area of the *H*-binding site is highlighted in A on the right top. Level of theory: APF-D/6-31G(d)

In Supporting Figure S1 (A) the 121 optimized structural geometries are shown by superimposition of phenol rings for all *H*-bonded **PH**···***pyr*** pairs. Consequently, the computed geometries can be displayed in a hemispherical-like interaction area, in which the pyridine migrates around the O−H group (Supporting Figure S1-A, right image highlights the nitrogen atom positions of the pyridine). In order to prove the suitability of the chosen method, the CA was recalculated using B97-D3/def2svp[16] level of theory (Supporting Figure S2).

Supporting Figure S1B and C show the evolution of the ZPE values depending on the scanning angle ζ(C−O···N) and dihedral angle Φ(C=C−O···N). Valleys, rendered in blue, represents the most stable conformers and shortest N···H bonds, respectively. Hilltops rendered in red brown represents unstable (not preferred) structures (Supporting Figure S1B) or alternatively longer N···H bonds (Supporting

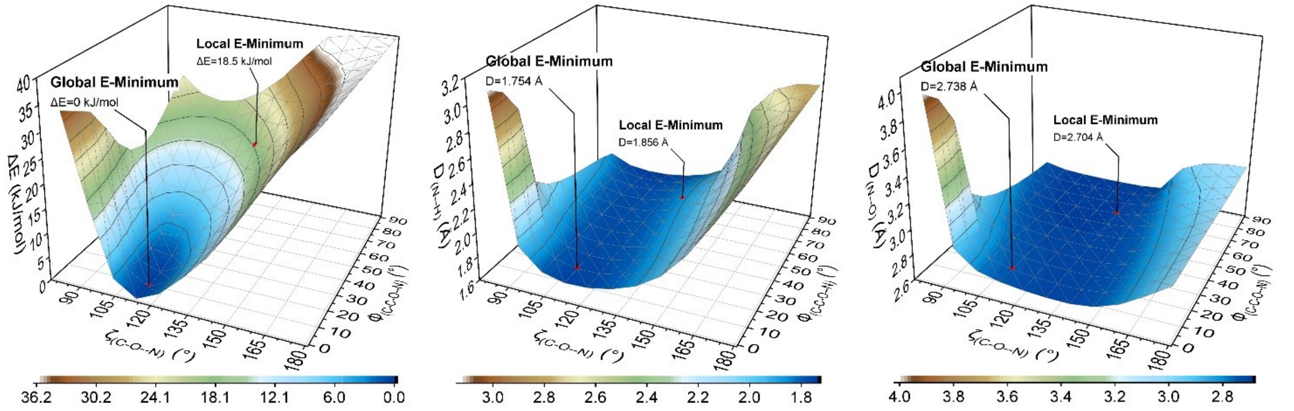

Supporting Figure S2. Relaxed potential energy surfaces computed from the conformational analysis of the **PH**···***pyr*** system shows the energetic change of the hydrogen bridge depending on the relative position of pyridine to the O–H group. Level of theory: B97-D3/def2svp.

Figure S1C). The calculated lowest energy **PH**···***pyr*** conformer is highlighted in green (Supporting Figure S1A), exhibiting the angles ζ ~113.5° and Φ ~18.0° and distance of the hydrogen bond of 1.798 Å (Supporting Figure S1B and C, Global E-Minimum). Changing these angles, for instance, to 132.5 and 62.0°, respectively, lead to an increase of the hydrogen bond length (D = 1.894 Å) and electronic energy (+15.8 kJ/mol) of the supramolecular system (Supporting Figure S1A–C, red structure, Local E-Minimum). Based on a pre-optimized geometry of the **PH**···***pyr*** system, its binding energy was calculated by -48.7 kJ/mol using APF-D/6-31G(d) level of theory. Correlating the relative position of ***pyr*** to the OH group with the optimized geometry revealed an increase in energy for the hydrogen bond of up to 36.2 kJ/mol. This is particularly reflected at small (ζ <100°) and large (ζ >150°) angles that can be explained by either repulsive interactions between ortho-substituted hydrogens in phenol and 2*H* in pyridine at small angles or stretching of the O–H bond to overcome the energy barrier of the hybridization. The same trend was also obtained for the change of dihedral angle Φ. These geometric changes were accompanied by an extension of the length of the hydrogen bonds in the range of 2.6–3.0 Å. In contrast, **PH**···***pyr*** conformers with binding angles for ζ = 100–135° and Φ = 0–30°, are energetically favored with short interaction distances in the a range of 1.8–2.0 Å, which are in line with distances found in crystalline structures of phenol-based assemblies.[17,18] The energy differences of hydrogen bonds for these conformers were found to be up to 6.0 kJ/mol (blue rendered region), which was insignificantly lower to aromatic COOH···***pyr*** interaction, where the pyridine moiety is anti-coplanarly arranged to the carboxylic acid group.[19] These results prove the high flexibility of the phenolic COH···***pyr*** binding motif and support a wide variety of geometries.

4 Computational Chemistry of the PHG···*pyr*$_3$ Auxiliary

4.1 Population Distribution Analysis

Using ΔG values computed at different temperatures we applied the Boltzmann weighting method to obtain the conformational population distribution average (fraction) of **PHG···*pyr*$_3$** conformers.[20]

First, we calculated the Boltzmann factor q_i (originally defined by Boltzmann[21] and Maxwell[22] according to Müller[23]) for each conformer at different temperatures using following equation:

$$q_i = e^{-\frac{\Delta G_i}{kT}}$$

where ΔG_i is the Gibbs free energy difference between conformer i and the lowest energy conformer, k is the Boltzmann constant and T is the treated temperature concerning Gibbs free energy computation. Second, the fraction of a conformer f_i can be expressed by equation:

$$f_i = \frac{q_i}{\sum_{j=1}^{3} q_j}$$

The sum $\sum_{j=1}^{3} q_j$ corresponds to the three conformers of **PHG···*pyr*$_3$** auxiliary (λ-, *E*- and *S*-shape).

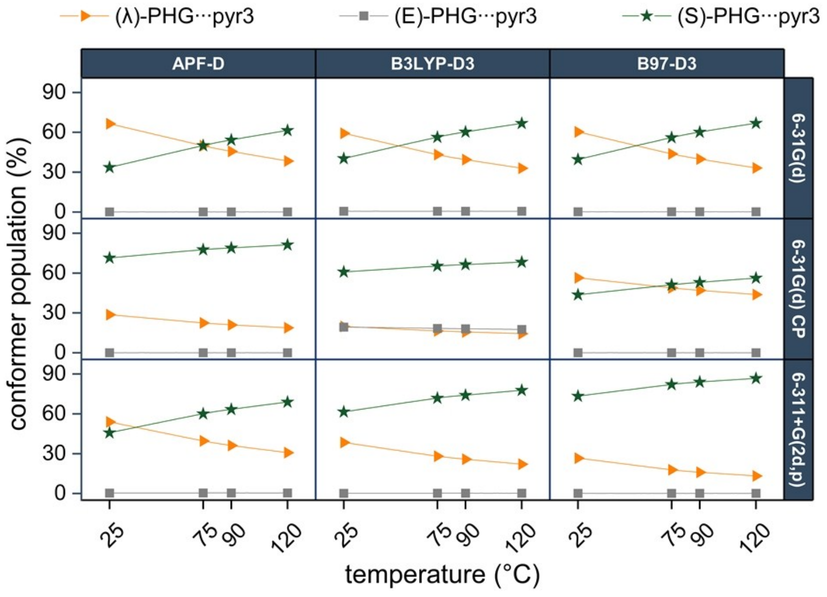

Supporting Figure S3. Temperature dependence of conformational population distribution (pop.) of **PHG···*pyr*$_3$** auxiliary using APF-D, B3LYP-D3 and B97-D3 methods with basis sets 6-31G(d), 6-31G(d) incl. CP correction and 6-311+G(2d,p).

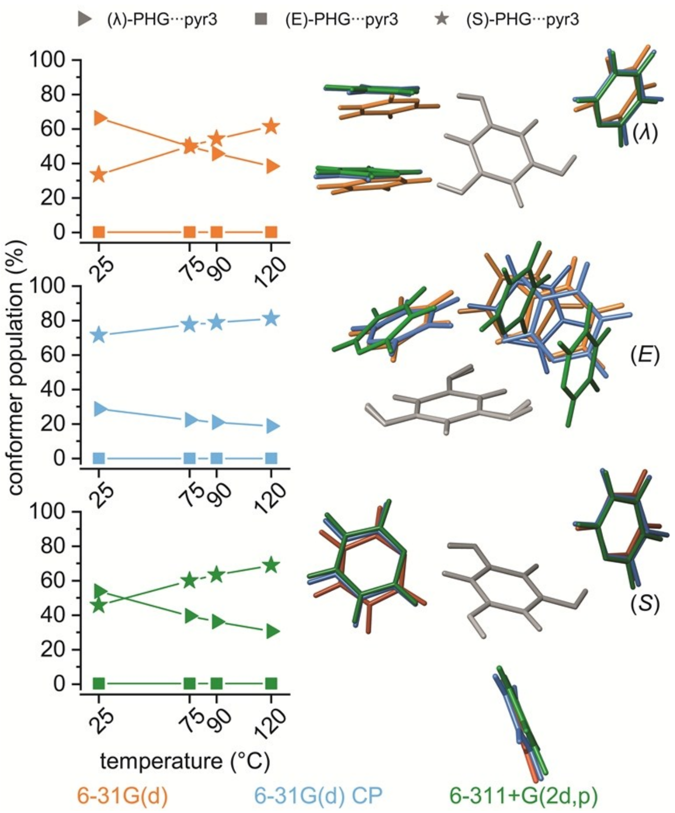

Supporting Figure S4. Superimposition of all the same conformers calculated at different level of theory shows a critical displacement of atoms for basis set 6-31G(d) without CP, while this virtually not obtained using CP and 6-311+G(2d,p).

Supporting Table S1. Calculated relative zero point energies (ΔZPE), Enthalpy (ΔH) and Gibbs free energies (ΔG) as well as weighted conformer population (pop.) of **PHG**···*pyr*$_3$ auxiliary using B3LYP-D3, B97-D3 and APF-D level of theory with basis sets 6-31G(d) with and without counterpoise correction (CP) as well as 6-311+G(2d,p). Gibbs free energies (ΔG) and weighted conformer population (pop.) have been computed regarding four different temperatures given.

		functional	B97-D3				B3LYP-D3				APF-D			
basis	temp.	conformer	ΔZPE (kJmol^{-1})	ΔH (kJmol^{-1})	ΔG (kJmol^{-1})	pop. (%)	ΔZPE (kJmol^{-1})	ΔH (kJmol^{-1})	ΔG (kJmol^{-1})	pop. (%)	ΔZPE (kJmol^{-1})	ΔH (kJmol^{-1})	ΔG (kJmol^{-1})	pop. (%)
6-31G(d)	25°C	(λ)-**PHG**···*pyr*$_3$	0.0	0.0	0.0	60.3	0.0	0.0	0.0	59.2	0.0	0.0	0.0	66.4
		(*E*)-**PHG**···*pyr*$_3$	3.0	2.5	14.0	0.2	5.7	5.2	11.8	0.5	3.8	3.5	15.2	0.1
		(*S*)-**PHG**···*pyr*$_3$	11.0	11.6	1.0	39.5	10.6	11.2	1.0	40.2	11.3	11.9	1.7	33.5
	75°C	(λ)-**PHG**···*pyr*$_3$	"	0.0	0.7	43.8	"	0.0	0.8	43.2	"	0.0	0.0	49.9
		(*E*)-**PHG**···*pyr*$_3$	"	2.7	16.6	0.2	"	5.3	13.6	0.5	"	3.7	17.2	0.1
		(*S*)-**PHG**···*pyr*$_3$	"	11.5	0.0	56.0	"	11.2	0.0	56.3	"	11.8	0.0	50.0
	90°C	(λ)-**PHG**···*pyr*$_3$	"	0.0	1.2	39.8	"	0.0	1.3	39.4	"	0.0	0.5	45.7
		(*E*)-**PHG**···*pyr*$_3$	"	2.7	17.7	0.2	"	5.3	14.5	0.5	"	3.8	18.2	0.1
		(*S*)-**PHG**···*pyr*$_3$	"	11.5	0.0	60.1	"	11.2	0.0	60.2	"	11.8	0.0	54.2
	120°C	(λ)-**PHG**···*pyr*$_3$	"	0.0	2.3	33.1	"	0.0	2.3	32.9	"	0.0	1.5	38.4
		(*E*)-**PHG**···*pyr*$_3$	"	2.8	19.9	0.2	"	5.4	16.2	0.5	"	3.9	20.4	0.1
		(*S*)-**PHG**···*pyr*$_3$	"	11.5	0.0	66.8	"	11.2	0.0	66.7	"	11.8	0.0	61.4
6-31G(d) CP	25°C	(λ)-**PHG**···*pyr*$_3$	0.0	0.0	0.0	56.4	0.0	0.0	2.8	19.8	0.0	0.0	2.3	28.6
		(*E*)-**PHG**···*pyr*$_3$	10.5	10.9	20.7	0.0	2.1	2.2	2.8	19.3	10.8	11.1	21.8	0.0
		(*S*)-**PHG**···*pyr*$_3$	5.1	5.2	0.6	43.3	4.2	4.5	0.0	60.9	5.4	5.6	0.0	71.4
	75°C	(λ)-**PHG**···*pyr*$_3$	"	0.0	0.1	48.8	"	0.0	4.0	16.4	"	0.0	3.6	22.4
		(*E*)-**PHG**···*pyr*$_3$	"	11.1	22.4	0.0	"	2.2	3.7	18.3	"	11.4	24.5	0.0
		(*S*)-**PHG**···*pyr*$_3$	"	5.2	0.0	51.1	"	4.4	0.0	65.3	"	5.6	0.0	77.6
	90°C	(λ)-**PHG**···*pyr*$_3$	"	0.0	0.4	47.0	"	0.0	4.4	15.6	"	0.0	4.0	21.0
		(*E*)-**PHG**···*pyr*$_3$	"	11.1	23.2	0.0	"	2.2	3.9	18.0	"	11.4	25.3	0.0
		(*S*)-**PHG**···*pyr*$_3$	"	5.2	0.0	53.0	"	4.4	0.0	66.4	"	5.6	0.0	78.9
	120°C	(λ)-**PHG**···*pyr*$_3$	"	0.0	0.8	43.8	"	0.0	5.1	14.4	"	0.0	4.8	18.8
		(*E*)-**PHG**···*pyr*$_3$	"	11.3	24.6	0.0	"	2.2	4.5	17.5	"	11.6	26.9	0.0
		(*S*)-**PHG**···*pyr*$_3$	"	5.2	0.0	56.2	"	4.4	0.0	68.2	"	5.5	0.0	81.2
6-311+G(2d,p)	25°C	(λ)-**PHG**···*pyr*$_3$	0.0	0.0	2.5	26.6	0.0	0.0	1.2	38.4	0.0	0.0	0.7	43.2
		(*E*)-**PHG**···*pyr*$_3$	7.4	7.4	17.3	0.1	10.0	10.2	15.4	0.1	7.6	7.7	13.2	0.3
		(*S*)-**PHG**···*pyr*$_3$	8.3	8.9	0.0	73.3	7.6	8.1	0.0	61.5	9.5	10.0	0.0	56.6
	75°C	(λ)-**PHG**···*pyr*$_3$	"	0.0	4.4	17.8	"	0.0	2.7	28.1	"	0.0	1.2	39.6
		(*E*)-**PHG**···*pyr*$_3$	"	7.6	20.5	0.1	"	10.3	17.6	0.2	"	7.9	14.5	0.4
		(*S*)-**PHG**···*pyr*$_3$	"	8.9	0.0	82.1	"	8.1	0.0	71.8	"	10.0	0.0	60.0
	90°C	(λ)-**PHG**···*pyr*$_3$	"	0.0	5.0	16.0	"	0.0	3.2	25.8	"	0.0	1.7	36.2
		(*E*)-**PHG**···*pyr*$_3$	"	7.6	21.4	0.1	"	10.4	18.3	0.2	"	7.9	15.2	0.4
		(*S*)-**PHG**···*pyr*$_3$	"	8.9	0.0	83.9	"	8.1	0.0	74.0	"	10.0	0.0	63.3
	120°C	(λ)-**PHG**···*pyr*$_3$	"	0.0	6.1	13.2	"	0.0	4.1	22.1	"	0.0	2.6	30.7
		(*E*)-**PHG**···*pyr*$_3$	"	7.7	23.3	0.1	"	10.5	19.6	0.2	"	8.0	16.6	0.4
		(*S*)-**PHG**···*pyr*$_3$	"	8.9	0.0	86.7	"	8.0	0.0	77.7	"	10.0	0.0	68.9

4.2 Geometric Parameters

Supporting Table S2. Geometric parameters of the H-bond motifs O–H···N for (*S*)-**PHG**···***pyr***$_3$. D denotes the bonding distance between the corresponding atoms, ζ the bonding angle for the H-bond and Φ describes the relative orientation of the O–H group with respect to the plane of the aromatic ring of **PHG** (dihedral angle).

functional	B97-D3			B3LYP-D3			APF-D		
basis set	6-31G (d)	6-31G (d) CP	6-311+G (2d,p)	6-31G (d)	6-31G (d) CP	6-311+G (2d,p)	6-31G (d)	6-31G (d) CP	6-311+G (2d,p)
H-bond group									
(O–H···N) motif*1*									
D(O–H) [Å]	0.994	0.994	0.993	0.987	0.986	0.985	0.987	0.987	0.986
D(O···N) [Å]	2.824	2.868	2.802	2.826	2.871	2.810	2.788	2.825	2.767
D(H···N) [Å]	1.857	1.890	1.819	1.866	1.897	1.833	1.831	1.854	1.794
ζ(O–H···N) [°]	163.1	167.3	170.0	163.1	168.5	170.4	162.3	167.1	168.3
Φ(C=C–O–H) [°]	12.9	14.0	13.6	12.0	13.2	12.2	12.1	14.5	14.5
(O–H···N) motif*2*									
D(O–H) [Å]	0.994	0.994	0.993	0.987	0.987	0.985	0.987	0.987	0.986
D(O···N) [Å]	2.823	2.869	2.803	2.825	2.873	2.811	2.786	2.826	2.767
D(H···N) [Å]	1.854	1.889	1.819	1.866	1.897	1.832	1.832	1.855	1.793
ζ(O–H···N) [°]	162.7	168.3	170.8	162.9	169.3	171.6	161.8	167.6	169.2
Φ(C=C–O–H) [°]	9.9	11.6	10.9	9.3	11.2	10.1	8.8	12.0	11.8
(O–H···N) motif*3*									
D(O–H) [Å]	0.994	0.994	0.993	0.987	0.987	0.986	0.987	0.987	0.986
D(O···N) [Å]	2.829	2.871	2.805	2.833	2.874	2.811	2.793	2.828	2.767
D(H···N) [Å]	1.860	1.889	1.819	1.868	1.896	1.831	1.833	1.855	1.792
ζ(O–H···N) [°]	164.0	168.9	171.7	164.8	170.2	172.5	163.4	168.2	170.6
Φ(C=C–O–H) [°]	10.7	10.6	10.0	10.5	9.7	8.9	10.1	11.3	10.8

Supporting Table S3. Geometric parameters of the H-bond motifs O–H···N for (*λ*)-**PHG**···***pyr***$_3$. D denotes the bonding distance between the corresponding atoms, ζ the bonding angle for the H-bond and Φ describes the relative orientation of the O–H group with respect to the plane of the aromatic ring of **PHG** (dihedral angle).

functional	B97-D3			B3LYP-D3			APF-D		
basis set	6-31G (d)	6-31G (d) CP	6-311+G (2d,p)	6-31G (d)	6-31G (d) CP	6-311+G (2d,p)	6-31G (d)	6-31G (d) CP	6-311+G (2d,p)
H-bond group									
(O–H···N) motif*1*									
D(O–H) [Å]	0.994	0.993	0.992	0.987	0.986	0.985	0.986	0.986	0.986
D(O···N) [Å]	2.823	2.871	2.803	2.823	2.875	2.810	2.785	2.828	2.769
D(H···N) [Å]	1.860	1.894	1.820	1.869	1.900	1.831	1.835	1.858	1.794
ζ(O–H···N) [°]	162.2	167.2	170.4	161.7	169.3	171.9	160.8	167.2	169.3
Φ(C=C–O–H) [°]	11.1	12.0	10.8	10.1	10.6	8.9	10.1	12.2	11.9
(O–H···N) motif*2*[a]									
D(O–H) [Å]	0.990	0.990	0.988	0.983	0.984	0.981	0.982	0.983	0.981
D(O···N) [Å]	2.865	2.913	2.855	2.865	2.917	2.864	2.829	2.871	2.821
D(H···N) [Å]	1.921	1.937	1.875	1.929	1.942	1.888	1.896	1.908	1.854
ζ(O–H···N) [°]	158.5	168.3	170.8	158.1	170.4	172.4	157.6	165.9	167.8
Φ(C=C–O–H) [°]	12.4	10.3	9.4	11.1	8.9	7.6	12.1	12.2	11.8
(O–H···N) motif*3*[a]									
D(O–H) [Å]	0.993	0.992	0.990	0.985	0.985	0.983	0.985	0.985	0.984
D(O···N) [Å]	2.817	2.901	2.842	2.811	2.908	2.851	2.779	2.853	2.802
D(H···N) [Å]	1.867	1.912	1.853	1.877	1.924	1.868	1.842	1.874	1.821
ζ(O–H···N) [°]	159.1	174.3	176.4	157.0	176.9	178.4	157.8	172.4	174.1
Φ(C=C–O–H) [°]	7.3	3.1	3.5	6.0	1.3	1.5	7.4	5.4	6.6

[a]parameters obtained from the two parallel side chains aligned in the head-to-head fashion.

Supporting Table S4. Geometric parameters of the H-bond motifs O–H···N for (*E*)-**PHG**···***pyr***$_3$. D denotes the bonding distance between the corresponding atoms, ζ the bonding angle for the H-bond and Φ describes the relative orientation of the O–H group with respect to the plane of the aromatic ring of **PHG** (dihedral angle).

functional	B97-D3			B3LYP-D3			APF-D		
basis set	6-31G (d)	6-31G (d) CP	6-311+G (2d,p)	6-31G (d)	6-31G (d) CP	6-311+G (2d,p)	6-31G (d)	6-31G (d) CP	6-311+G (2d,p)
H-bond group									
(O–H···N) motif*1*									
D(O–H) [Å]	0.981	0.975	0.976	0.982	0.986	0.972	0.974	0.970	0.971
D(O···N) [Å]	2.968	3.323	2.977	2.835	2.875	2.964	2.924	3.168	2.925
D(H···N) [Å]	2.219	2.743	2.151	1.985	1.897	2.141	2.181	2.535	2.103
ζ(O–H···N) [°]	132.2	118.7	141.4	143.4	170.7	141.5	132.0	123.0	141.4
Φ(C=C–O–H) [°]	33.9	20.0	32.9	25.7	8.6	32.9	34.1	24.1	33.4
(O–H···N) motif*2*[a]									
D(O–H) [Å]	0.993	0.993	0.986	0.983	0.984	0.979	0.985	0.986	0.979
D(O···N) [Å]	2.796	2.851	2.855	2.806	2.916	2.857	2.764	2.809	2.821
D(H···N) [Å]	1.869	1.885	1.917	1.935	1.949	1.927	1.850	1.855	1.891
ζ(O–H···N) [°]	154.1	163.3	158.1	146.2	167.0	157.5	153.0	162.1	157.5
Φ(C=C–O–H) [°]	12.7	12.4	20.1	28.4	6.7	19.9	11.2	11.8	18.9
(O–H···N) motif*3*[a]									
D(O–H) [Å]	0.989	0.990	0.992	0.985	0.984	0.985	0.982	0.982	0.985
D(O···N) [Å]	2.861	2.897	2.818	2.808	2.901	2.825	2.828	2.859	2.787
D(H···N) [Å]	1.942	1.954	1.830	1.889	1.928	1.845	1.918	1.927	1.807
ζ(O–H···N) [°]	153.3	158.4	174.2	154.0	169.4	173.4	153.0	157.4	172.7
Φ(C=C–O–H) [°]	19.2	17.3	4.1	8.7	4.3	3.9	18.5	17.8	4.4

[a]parameters obtained from the two parallel side chains aligned in the head-to-head fashion.

4.3 Vibrational Modes

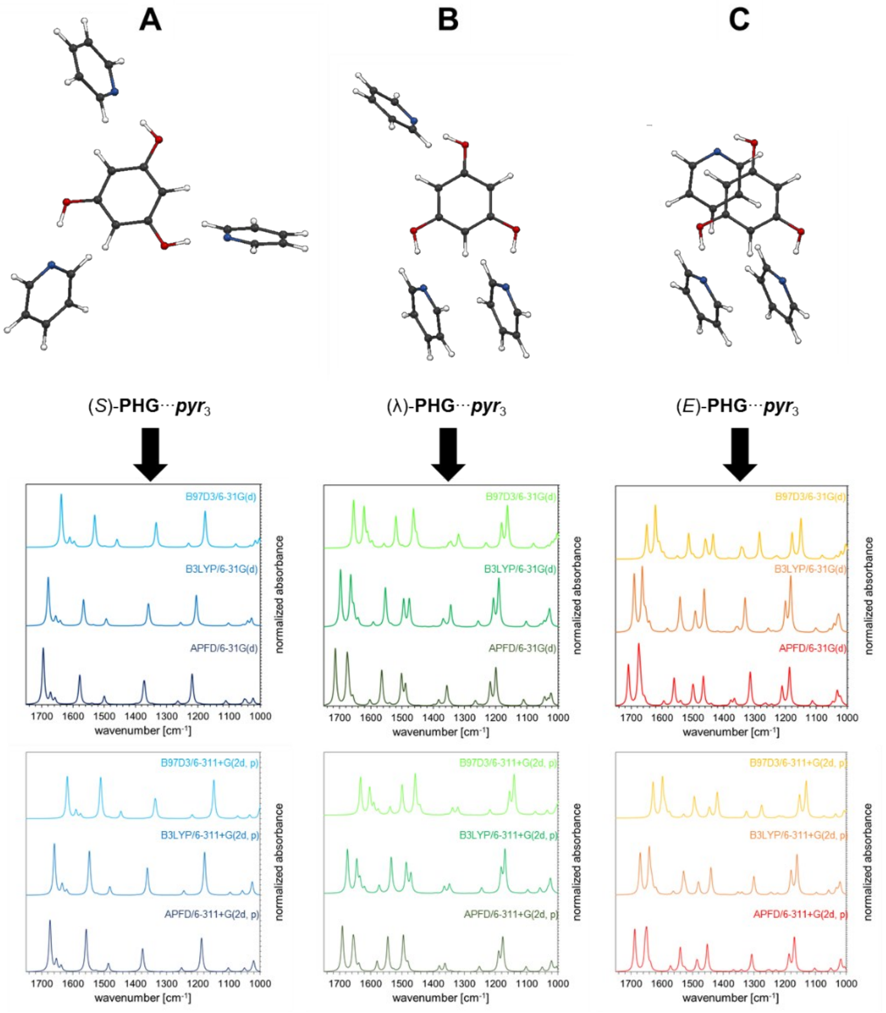

Supporting Figure S5. Vibrational frequency calculations using different level of theories for the star (A), lambda (B) and *E*-shaped (C) conformations.

5 Characteristic Values within the PHG···(Ap-8)3 System

5.1 Electronic Properties and calculated Energies

Supporting Table S5. Dipole moment (μ) and polarizability (α) as well as absolute complexation energy (E_C), relative zero point energies (ΔZPE), enthalpy (ΔH), Gibbs free energies (ΔG) of monomeric **PHG**···(**Ap**-***8***)$_3$ and weighted conformer population distribution (pop.) at 25, 90 and 120 °C using APF-D/6-31G(d) level of theory with counterpoise correction.

		μ (D)	α (a.u.)	E_C (kJ/mol)	ΔZPE ($kJmol^{-1}$)	ΔH ($kJmol^{-1}$)	ΔG ($kJmol^{-1}$)	pop. (%)
25°C	(λ)-**PHG**···(**Ap**-***8***)$_3$	11.6	877.9	-213.8	57.0	60.9	10.7	1.3
	(*E*)-**PHG**···(**Ap**-***8***)$_3$	21.8	805.7	-302.4	0.00	0.0	0.0	98.7
	(*S*)-**PHG**···(**Ap**-***8***)$_3$	2.2	918.6	-143.6	113.3	120.4	25.1	0.0
90°C	(λ)-**PHG**···(**Ap**-***8***)$_3$	"	"	"	"	60.8	0.0	46.8
	(*E*)-**PHG**···(**Ap**-***8***)$_3$	"	"	"	"	0.0	0.3	42.8
	(*S*)-**PHG**···(**Ap**-***8***)$_3$	"	"	"	"	120.3	4.6	10.4
120°C	(λ)-**PHG**···(**Ap**-***8***)$_3$	"	"	"	"	60.8	0.0(0)	45.7
	(*E*)-**PHG**···(**Ap**-***8***)$_3$	"	"	"	"	0.0	5.3	9.0
	(*S*)-**PHG**···(**Ap**-***8***)$_3$	"	"	"	"	120.3	0.0(2)	45.4

Supporting Table S6. Dipole moment (μ) and polarizability (α) as well as absolute complexation energy (E_C), relative zero point energies (ΔZPE), enthalpy (ΔH), Gibbs free energies (ΔG) of monomeric **PHG**···(**Ap**-***8***)$_3$ and weighted conformer population distribution (pop.) at 25°C using B97-D3/def2svp level of theory with counterpoise correction.

		μ (D)	α (a.u.)	E_C (kJ/mol)	ΔZPE ($kJmol^{-1}$)	ΔH ($kJmol^{-1}$)	ΔG ($kJmol^{-1}$)	pop. (%)
25°C	(λ)-**PHG**···(**Ap**-***8***)$_3$	11.8	1318.4	-201.4	49.0	60.9	9.3	2.3
	(*E*)-**PHG**···(**Ap**-***8***)$_3$	22.5	956.4	-280.4	0.0	0.0	0.0	97.7
	(*S*)-**PHG**···(**Ap**-***8***)$_3$	1.8	1257.8	-137.6	101.2	120.4	26.5	0.0

Supporting Table S7. Relative zero point energy (ΔZPE), enthalpy (ΔH) and Gibbs free energies (ΔG) of the dimeric **PHG**···(**Ap**-***8***)$_3$ obtained at 25 °C using APF-D/6-31G(d) level of theory.

	μ (D)	α (a.u.)	ΔZPE ($kJmol^{-1}$)	ΔH ($kJmol^{-1}$)	ΔG ($kJmol^{-1}$)
(dλ)-**PHG**···(**Ap**-***8***)$_3$	25.0	1717.0	130.9	136.3	86.4
(d*E*)-**PHG**···(**Ap**-***8***)$_3$	42.5	1649.5	0.00	0.0	0.0
(d*S*)-**PHG**···(**Ap**-***8***)$_3$	2.7	1809.5	153.3	159.2	101.4

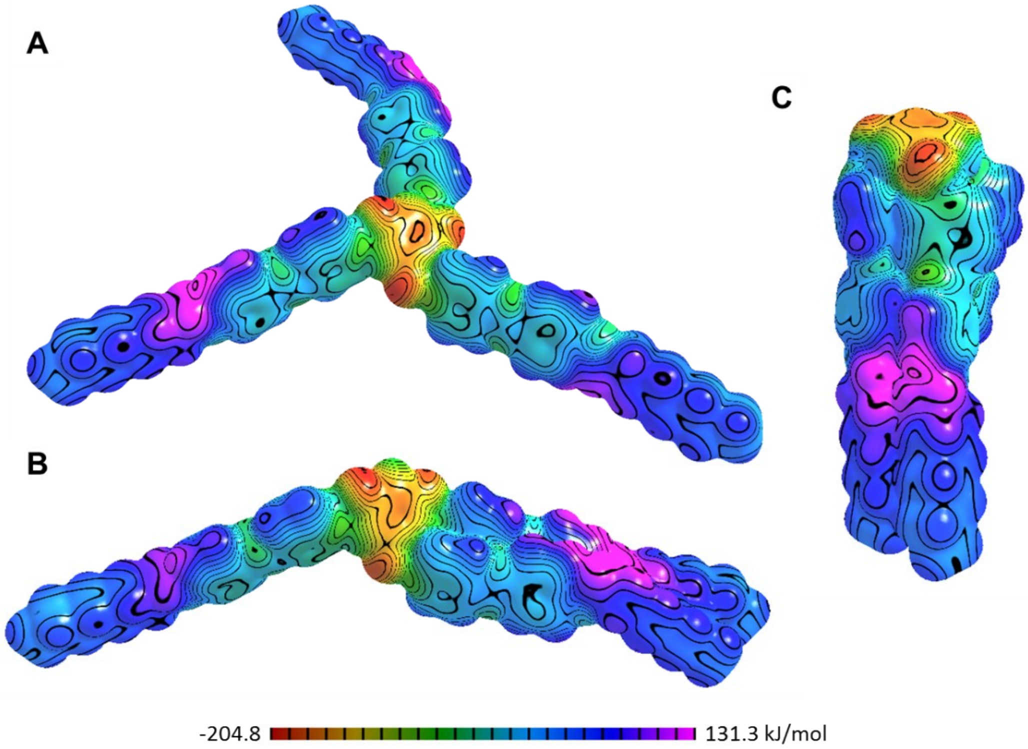

Supporting Figure S6. ESP isosurface of the three **PHG** conformers, visualizing best why the *E*-folded structure exhibit the highest dipole moment among the conformers of **PHG**···(**Ap**-***8***)$_3$ assemblies.

5.2 Geometric Parameters

Supporting Table S8. Geometric parameters of the hydrogen bonding motifs O–H···N using APF-D/6-31G(d)-CP level of theory. D denotes the bonding length between the corresponding atoms, ζ the bond angle at the hydrogen bond and Φ describes the relative orientation of the O–H group with respect to the plane of the aromatic ring of **PHG** (dihedral angle).

	(*S*)-<br>**PHG**···(**Ap**-***8***)$_3$	(d*S*)-<br>**PHG**···(**Ap**-***8***)$_3$[a]	(λ)-<br>**PHG**···(**Ap**-***8***)$_3$	(dλ)-<br>**PHG**···(**Ap**-***8***)$_3$[a]	(*E*)-<br>**PHG**···(**Ap**-***8***)$_3$	(d*E*)-<br>**PHG**···(**Ap**-***8***)$_3$[a,c]
motif 1						
D(O–H) (Å)	0.986	0.987|0.992	0.986	0.988 | 0.985	0.978	0.984 | 0.988
D(O···N) (Å)	2.824	2.795 | 2.745	2.828	2.756 | 2.829	2.842	2.776 | 2.817
D(H···N) (Å)	1.852	1.815 | 1.770	1.857	1.812 | 1.868	2.045	1.897 | 1.863
ζ(O–H···N) (°)	167.7	171.9 | 166.5	167.3	158.6 | 164.4	137.2	147.1 | 161.1
Φ(C=C–O–H) (°)	-12.2	-14.6 | -11.8	-13.1	-12.8 | -24.3	34.5	30.1 | 44.6
motif 2[b]						
D(O–H) (Å)	0.986	0.988 | 0.987	0.984	0.982 | 0.985	0.984	0.983 | 0.983
D(O···N) (Å)	2.823	2.752 | 2.801	2.858	2.795 | 2.801	2.828	2.818 | 2.817
D(H···N) (Å)	1.854	1.813 | 1.828	1.886	1.855 | 1.842	1.912	1.933 | 1.940
ζ(O–H···N) (°)	166.6	157.4 | 167.7	168.6	159.1 | 163.5	153.7	148.3 | 147.3
Φ(C=C–O–H) (°)	14.7	11.2 | 12.6	12.6	3.17 | 7.51	23.8	42.2 | 44.5
motif 3[b]						
D(O–H) (Å)	0.986	0.985 | 0.984	0.985	0.981 | 0.981	0.984	0.984 | 0.986
D(O···N) (Å)	2.824	2.776 | 2.830	2.845	2.878 | 2.835	2.779	2.738 | 2.733
D(H···N) (Å)	1.850	1.845 | 1.881	1.869	1.936 | 1.902	1.883	1.857 | 1.853
ζ(O–H···N) (°)	168.6	156.3 | 160.8	170.7	160.2 | 157.9	149.9	147.4 | 146.9
Φ(C=C–O–H) (°)	-11.2	-23.7 | -6.33	5.40	13.1 | 12.3	27.2	42.0 | 41.7

[a]the pairs of values (left|right) were excerpted out of the same symmetric **Ap**. The values listed on one site belongs to the same assembly.
[b]count only for the λ- and *E*-folded assemblies: parameters obtained from the two parallel side chains aligned in the head-to-head fashion.
[c]left values are part of the upper **PHG** assembly.

5.3 Complexation and Interaction Energies

A comparison of the conformational stabilities, complexation (E_C) and interaction energies (ΔE_{int}) as well as BSSE were calculated by single point energy calculations using APF-D/6-31G(d) CP level of theory. ΔE_{int} has been calculated from the differences of E_C of the corresponding hydrogen-bonded systems. The green-labelled conformers highlight assemblies, which consists of *O*-methyl groups (truncated model system).

Supporting Table S9. Absolute and relative energies of (*S*)-**PHG**···(**Ap**-***8***)$_3$.

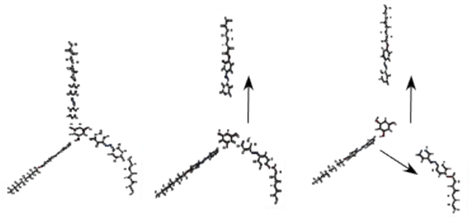

E_C (kJmol^{-1})	-143.51	-97.86	-50.62
ΔE_{int} (kJ/mol)	0.00	-45.65	-47.24
BSSE (kJmol^{-1})	25.43	16.85	8.38

Supporting Table S10. Absolute and relative energies of (λ)-**PHG**···(**Ap**-***8***)$_3$.

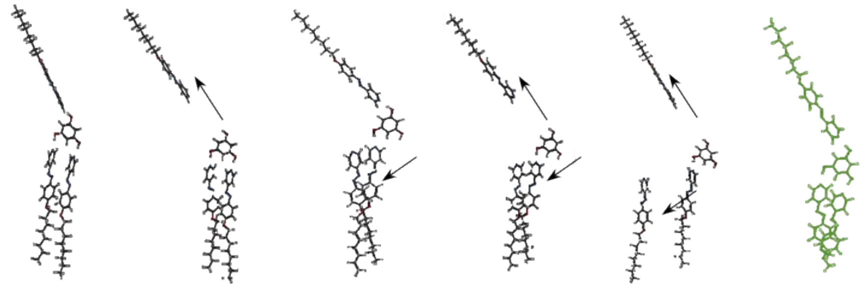

E_C (kJmol^{-1})	-213.64	-169.41	-170.00	-123.22	-48.41	-187.99
ΔE_{int} (kJ/mol)	0.00	-44.22	-43.64	-46.78	-74.81	-25.65
BSSE (kJmol^{-1})	47.31	38.88	39.78	31.30	8.54	42.39

Supporting Table S11. Absolute and relative energies of (*E*)-**PHG**···(**Ap**-***8***)$_3$.

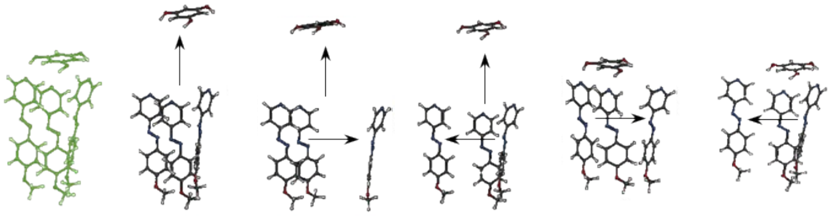

E_C (kJmol^{-1})	-234.18	-105.98	-41.63	-37.03	-133.68	-120.62
ΔE_{int} (kJ/mol)	0.00	-128.20	-64.35	-68.95	-100.50	-113.56
BSSE (kJmol^{-1})	63.92	32.68	14.30	11.22	34.51	32.69

5.4 Vibrational Modes

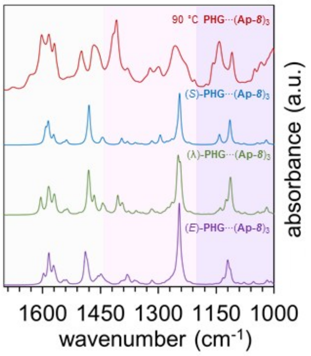

Supporting Table S12. Wavenumbers (cm^{-1}) calculated as well as vibrational modes of **PHG**···(**Ap**-***8***)$_3$ in their polymorphic forms (*S*, λ, *E*). Calculations were performed using APF-D/6-31G(d) level of theory. ϑ_s: symmetric stretching; ρ: rocking; β: in-the-plane bending; *R*: ring deformation. Scale factor used for the assembly and cluster structures: 0.94.

**PHG**···(**Ap**-***8***)$_3$ practical	scale factor: 0.940 *S*-form		0.940 λ-form		0.940 *E*-form	
	1592.48	β*R*(**PHG**)	1604.76	β*R*(**PHG**)	1597.69	β*R*(**PHG**)
		β(O20–H21)		β(O20–H21)		β(O20–H21)
		β(O24–H25)		β(O22–H23)		β(O22–H23)
	1591.88	β*R*(**PHG**)		β(O24–H25)		β(O24–H25)
		β(O20–H21)	1585.54	β*R*(ph$_{N6}$)	1583.93	β*R*(ph$_{N6}$)
		β(O22–H23)		β*R*(ph$_{N69}$)		β*R*(ph$_{N69}$)
		β(O24–H25)	1582.75	β*R*(ph$_{N6}$)		β*R*(ph$_{N117}$)
	1585.16	β*R*(ph$_{N6}$)		β*R*(ph$_{N69}$)	1583.57	β*R*(ph$_{N6}$9)
		β*R*(ph$_{N69}$)	1581.86	β*R*(ph$_{N117}$)		β*R*(ph$_{N117}$)
		β*R*(ph$_{N117}$)	1571.34	β*R*(**PHG**)	1581.82	β*R*(ph$_{N6}$)
	1585.15	β*R*(ph$_{N69}$)		β(O20–H21)		β*R*(ph$_{N69}$)
		β*R*(ph$_{N117}$)		β(O24–H25)		β*R*(ph$_{N117}$)
	1571.61,	β*R*(***pyr***$_{N6}$)	1568.81	β*R*(***pyr***$_{N6}$)	1572.28	β*R*(***pyr***$_{N6}$)
	1571.23	β*R*(***pyr***$_{N69}$)		β*R*(***pyr***$_{N69}$)		β*R*(**PHG**)
		β*R*(***pyr***$_{N117}$)	1567.78	β*R*(***pyr***$_{N117}$)		β(O22–H23)
	1571.14	β*R*(***pyr***$_{N69}$)	1566.30	β*R*(**PHG**)		β(O24–H25)
		β*R*(***pyr***$_{N117}$)		β(O20–H21)	1570.30	β*R*(**PHG**)
	1484.84	β*R*(**PHG**)		β(O24–H25)		β(O22–H23)
		β(O20–H21)		β*R*(***pyr***$_{N6}$)		β(O24–H25)
		β(O24–H25)		β*R*(***pyr***$_{N69}$)		β*R*(ph$_{N6}$)
	1484.54	β*R*(**PHG**)	1481.61	β*R*(ph$_{N6}$)		β*R*(ph$_{N69}$)
		β(O20–H21)		β*R*(ph$_{N69}$)	1566.87	β*R*(***pyr***$_{N6}$)
		β(O22–H23)		ϑs(N□N)N6	1565.53	βR(pyrN117)
		β(O24–H25)		ϑs(N□N)N69	1492.39	βR(PHG)
	1479.79	βR(PHG)	1479.04	βR(phN117)		β(O20–H21)
		β(O20–H21)		ϑs(N□N)N117		β(O22–H23)
		β(O24–H25)	1477.01	βR(phN6)		β(O24–H25)
		βR(phN69)		βR(phN69)	1489.46	βR(phN6)
		βR(phN117)		ϑs(N□N)N6	1484.08	βR(phN69)
	1479.67	βR(PHG)		ϑs(N□N)N69		βR(pyrN69)
		β(O20–H21)	1466.16	βR(PHG)	1480.68	βR(phN117)
		β(O22–H23)		β(O20–H21)		βR(pyrN117)

	β(O24–H25)		β(O22–H23)	1245.87	βR(aliph69)
1295.64	βR(**PHG**)		β(O24–H25)	1132.79	ρ(C11–H17)
	β(O20–H21)	1250.38	$\beta R(\mathrm{ph}_{N6})$		ρ(C13–H18)
	β(O22–H23)		$\beta R(\mathrm{ph}_{N69})$		ρ(C15–H19)
	β(O24–H25)	1248.08	$\beta R(\mathrm{ph}_{N6})$	1120.76	$\beta R(\mathrm{ph}_{N117})$
1294.56	β(O20–H21)		$\beta R(\mathrm{ph}_{N6}9)$	1118.58,	$\beta R(\mathrm{ph}_{N69})$
	β(O24–H25)	1242.73	$\beta R(\mathrm{ph}_{N117})$	1118.20	
1245.28	$\beta R(\mathrm{ph}_{N6})$	1147.82	$\beta R(\boldsymbol{pyr}_{N6})$	1113.54	$\beta R(\mathrm{ph}_{N6})$
	$\beta R(\mathrm{ph}_{N117})$		$\beta R(\boldsymbol{pyr}_{N69})$	1112.99	ρ(C11–H17)
1244.22	$\beta R(\mathrm{ph}_{N69})$		$\beta R(\mathrm{ph}_{N6})$		ρ(C13–H18)
1141.87	ρ(C11–H17)		$\beta R(\mathrm{ph}_{N69})$		
	ρ(C13–H18)	1147.06	$\beta R(\boldsymbol{pyr}_{N6})$		
	ρ(C15–H19)		$\beta R(\boldsymbol{pyr}_{N69})$		
1141.30	ρ(C13–H18)		$\beta R(\mathrm{ph}_{N6})$		
	ρ(C15–H19)		$\beta R(\mathrm{ph}_{N69})$		
1115.07,	$\beta R(\mathrm{ph}_{N6})$	1144.68	$\beta R(\boldsymbol{pyr}_{N117})$		
1114.48,	$\beta R(\mathrm{ph}_{N69})$		$\beta R(\mathrm{ph}_{N117})$		
1114.19	$\beta R(\mathrm{ph}_{N117})$	1139.58	ρ(C11–H17)		
			ρ(C15–H19)		
		1124.64	ρ(C13–H18)		
		1114.55	$\beta R(\mathrm{ph}_{N6})$		
			$\beta R(\mathrm{ph}_{N69})$		
		1112.03	$\beta R(\mathrm{ph}_{N117})$		
		1110.31	$\beta R(\mathrm{ph}_{N6})$		
			$\beta R(\mathrm{ph}_{N69})$		

6 References

(1) Gaussian 16, Revision A.03, M. J. Frisch, G. W. Trucks, H. B. Schlegel, G. E. Scuseria, M. A. Robb, J. R. Cheeseman, G. Scalmani, V. Barone, G. A. Petersson, H. Nakatsuji, X. Li, M. Caricato, A. Marenich, J. Bloino, B. G. Janesko, R. Gomperts, B. Mennucci, H. P. Hratchian, J. V. Ortiz, A. F. Izmaylov, J. L. Sonnenberg, D. Williams-Young, F. Ding, F. Lipparini, F. Egidi, J. Goings, B. Peng, A. Petrone, T. Henderson, D. Ranasinghe, V. G. Zakrzewski, J. Gao, N. Rega, G. Zheng, W. Liang, M. Hada, M. Ehara, K. Toyota, R. Fukuda, J. Hasegawa, M. Ishida, T. Nakajima, Y. Honda, O. Kitao, H. Nakai, T. Vreven, K. Throssell, J. A. Montgomery, Jr., J. E. Peralta, F. Ogliaro, M. Bearpark, J. J. Heyd, E. Brothers, K. N. Kudin, V. N. Staroverov, T. Keith, R. Kobayashi, J. Normand, K. Raghavachari, A. Rendell, J. C. Burant, S. S. Iyengar, J. Tomasi, M. Cossi, J. M. Millam, M. Klene, C. Adamo, R. Cammi, J. W. Ochterski, R. L. Martin, K. Morokuma, O. Farkas, J. B. Foresman, and D. J. Fox, Gaussian, Inc., Wallingford CT, 2016.

(2) Grimme, S., Ehrlich, S., Goerigk, *J. Comput. Chem.*, **2011**, *32*, 1456–1465.

(3) Grimme, S., Antony, J., Ehrlich, S., Krieg, H., *J. Chem. Phys.*, **2010**, *132*, 154104.

(4) Grimme, S., *J. Comput. Chem.*, **2006**, *27*, 1787–1799.

(5) Becke, A. D., *J. Chem. Phys.*, **1993**, *98*, 5648–5652.

(6) Stephens, P. J., Devlin, F. J., Chabalowski, C. F., Frisch, M. J, *J. Phys. Chem.* **1994**, *98*, 11623–11627.

(7) Austin, A., Petersson, G. A., Frisch, M. J., Dobek, F. J., Scalmani, G., Throssell, K., *J. Chem. Theory Comput.*, **2012**, *8*, 4989–5007.

(8) Hariharan, P. C., Pople, J. A. The influence of polarization functions on molecular orbital hydrogenation energies. *Theor. Chem. Acc.* **1973**, *28*, 213–222.

(9) Boys, S. F., Bernardi, F., *Mol. Phys.*, **1970**, *19*, 553–566.

(10) Simon, S., Duran, M., Dannenberg, J. J., *J. Chem. Phys.*, **1996**, *105*, 11024–11031.

(11) GaussView, Version 6, Roy Dennington, Todd A. Keith, and John M. Millam, Semichem Inc., Shawnee Mission, KS, 2016.

(12) Macrae, C. F., Edgington, P. R., McCabe, P., Pidcock, E., Shields, G. P., Taylor, R., Towler, M., van de Streek, J., *J. Appl. Crystallogr.*, **2006**, *39*, 453–457.

(13) Hübschle, C. B., Dittrich, B., *J. Appl. Crystallogr.*, **2011**, *44*, 238–240.

(14) Bader, R. F. W., Carroll, M. T., Cheeseman, J. R., Chang, C., *J. Am. Chem. Soc.*, **1987**, *109*, 7968–7979.

(15) Schrödinger Release 2018-4: Maestro, Schrödinger, LLC, New York, NY, 2018.

(16) Weigend, F., Ahlrichs, R., *Phys. Chem. Chem. Phys.*, **2005**, *7*, 3297–3305.

(17) Pfletscher, M., Wölper, C., Gutmann, J. S., Mezger, M., Giese, M., *Chem. Commun.*, **2016**, *52*, 8549–8552.

(18) Giese, M., Krappitz, T., Dong, R. Y., Michal, C. A., Hamad, W. Y., Patrick, B. O., MacLachlan, M. J., *J. Mater. Chem. C*, **2015**, *3*, 1537–1545.

(19) Fedorov, M. S., Giricheva, N. I., Shpilevaya, K. E., Lapykina, E. A., Syrbu, S. A., *J. Mol. Struct.*, **2017**, *1132*, 50–55.

(20) Wysoglad, J., Ehlers, J.-E., Lewe, T., Dornbusch, M., Gutmann, J. S., *J. Mol. Struct.*, **2018**, *1166*, 456–469.

(21) Boltzmann, L. *Studien über das Gleichgewicht der lebendigen Kraft zwischen bewegten materiellen Punkten*, Wien, 1868.

(22) Maxwell, J. C. *On Boltzmann's Theorem on the Average Distribution of Energy in a System of Material Points*, Printed at the University Press, and sold by Deighton, Bell and Company and Macmillan and Company Cambridge, Bell and Sons, London, 1879.

(23) Müller, I., *A history of thermodynamics: The doctrine of energy and entropy*, Springer: Berlin Heidelberg, 2007.
